# Phytochemical analysis, antimicrobial and antioxidant activities of *Euphorbia golondrina* L.C. Wheeler (Euphorbiaceae Juss.): an unexplored medicinal herb reported from Cameroon

**DOI:** 10.1186/s40064-016-1928-8

**Published:** 2016-03-02

**Authors:** Lawrence Monah Ndam, Afui Mathias Mih, Aaron Suh Tening, Augustina Genla Nwana Fongod, Nkegua Anna Temenu, Yoshiharu Fujii

**Affiliations:** Department of Botany and Plant Physiology, University of Buea, P.O. Box 63, Fako, South West Region Cameroon; Faculty of Agriculture and Veterinary Medicine, University of Buea, P.O. Box 63, Fako, South West Region Cameroon; ABA Home Health Care Inc., 821 Kennedy Street NW, Washington, DC 20011 USA; Department of International Environmental and Agricultural Sciences, Tokyo University of Agriculture and Technology, Fuchu Campus, Saiwai-cho, Fuchu, Tokyo 183-8509 Japan

**Keywords:** *Euphorbia golondrina*, Antioxidant activity, Antimicrobial activity, Phytochemicals, GC–MS

## Abstract

This study aimed at determining the phytochemical constituents of *Euphorbia golondrina* L.C. Wheeler, an alien invasive medicinal herb that is used for the treatment of gastroenteritis related ailments, diabetes, conjunctivitis, gastritis, enterocolitis, tonsillitis, vaginitis, hemorrhoids, prostatism, warts and painful swellings by the Mundani people of the mount Bambouto Caldera in SouthWestern Cameroon, and to evaluate its in vitro antimicrobial and antioxidant activities. Susceptibility testing by agar well diffusion assay revealed good antibacterial activity with inhibition zone diameter of 20 ± 1.1 mm against *Bacillus cereus* followed by *Staphylococcus aureus* with inhibition zone diameter of 17 ± 1.6 mm which was significantly lower (P < 0.05) than the positive control (amoxicillin). None of the fungi was inhibited by the acetone extract of *E. golondrina* except *Candida albicans* wherein the zone of inhibition was not significantly different from that of the positive control (Amphotericin B). The ABTS scavenging activity of *E. golondrina* was higher than that of gallic acid and BHT at concentrations greater than 0.1 and 0.2 mg/mL respectively while at all concentrations, nitric oxide scavenging activity was higher than those of both rutin and vitamin C. GC–MS profile of *E. golondrina* steam distilled volatiles revealed that the plant has potent phytoconstituent classes such as sesquiterpenes, monoterpenes, alkaloids, phenolics and aromatic hydrocarbons. Among the 30 compounds identified, caryophyllene oxide (14.16 %), camphor (9.41 %) and phytol (5.75 %) were the major compounds. Further structural characterisation based on ^1^H and ^13^C NMR is required to demonstrate structural integrity including correct stereochemistry. The current study partially justifies the ethnomedicinal uses of *E. golondrina* in Cameroon.

## Background

Increasing trends of microbial resistance to antibiotics and various chronic and degenerative pathologies of humans caused by reactive oxygen species(ROS) have triggered the search for bioactive compounds from plants with alternative mechanisms of action to counteract pathogenic microbes and natural antioxidants capable of protecting the body against oxidative stress and free radical-induced damage (Lu et al. 2007; Mbwambo et al. 2007; N’guessan et al. 2007; Newman and Cragg 2007; Duracková 2010; Reuter et al. 2010; Davies and Davies 2010; Buffet-Bataillon et al. 2012). The plant kingdom habours enormous amounts of therapeutic agents that have diverse applications in the pharmaceutical, nutraceutical and agrochemical industries. The active principles responsible for the therapeutic effects of medicinal plants are phytochemicals, usually secondary metabolites, including but not limited to alkaloids, steroids, flavonoids, terpenoids and tannins (NONITA et al. 2010). Of the reported 500 000 species of higher plants, only about 6 and 15 % have been evaluated for biological activity and phytochemical analysis respectively (Fabricant and Farnsworth 2010; Verpoorte 2000). Over 50 % of drugs are of plant origin (Balandrin et al. 1993). The tedious process of plant–based discovery is often based on knowledge of ethnopharmacopoeia of the plant. Such medicinal plants are screened in vitro for bioactivity using several techniques. Further identification of the lead compounds is based on the diversity of secondary metabolites or phytochemicals produced by the plants.

*Euphorbia golondrina* L.C. Wheeler (Syn. *Chamaesyce golondrina* (L.C. Wheeler) Shinners) belongs to the Euphorbiaceae family and the subgenus Chamaesyce. It is an herbaceous weedy plant found in the USA, Mexico and Cameroon (Ndam et al. 2015a). In the African ethnopharmacopoeia, it is known to treat gastroenteritis related ailments (Ndam et al. 2015a). An infusion of the roots is used in the management of diabetes while a decoction of the leaves mixed with *Senna alata* is exploited in the treatment of conjunctivitis, gastritis, enterocolitis, tonsillitis, vaginitis, hemorrhoids, prostatism and snake poison (Aleksandroff 2011; Rodriguez, 2013). The white latex from the stem is employed as an ointment in the treatment of warts and painful swellings by the Mundani people of the mount Bambouto Caldera, SouthWestern Cameroon (Ndam et al. 2015b). There is no information on the bioactivity and the phytochemical composition of this plant. Therefore in this study, the antimicrobial activity of *E. golondrina* against a panel of standardized pathogenic fungi and bacteria and the antioxidant properties of the acetone crude extract of the plant were elucidated. The phytochemical screening of the plant was also done to identify the secondary metabolites occurring in it.

## Methods

### Plant and preparation of crude extract

The *E. golondrina* earlier identified (Ndam et al. 2014, 2015a) was obtained in 2015 from the mount Bambouto caldera, SouthWestern Cameroon. The fresh leaves of the plant were cut, surface sterilised with 70 % alcohol and rinsed with sterile distilled water. The leaves were air-dried at room temperature for one month, mechanically ground to a fine powder by an electrical blender and further air-dried for 3 days. One hundred grams (100 g) of the powder was macerated in 1000 mL of acetone for 48 h three times under room temperature (22–25 °C). After filtering the resultant solution through a Whatman No. 1 grade filter paper, the filtrate was then concentrated to dryness under pressure at a maximum of 40 °C using a rotary evaporator (BUCHI Rotavapor R-200, Switzerland). The concentrate was recovered with a minimum volume of dichloromethane and kept open at room temperature until all the residual solvent had evaporated. The dried crude extract was weighed, and kept in a bottle sealed with parafilm and stored at 4 °C until used. An aliquot of the extract was resuspended in acetone to yield a 100 mg/mL stock solution (Koduru et al. 2006). The yield (%, w/w) of the dried extract was calculated as: Yield (%) = (W1 × 100)/W2, where W1 is the weight of the extract after lyophilization of solvent, and W2 is the weight of the plant powder.

### Microorganisms and antimicrobial susceptibility assays

The microbes used in this study were three Gram-positive bacteria: *Staphylococcus aureus*, *Enterococcus faecalis* and *B. cereus* and one Gram-negative bacterium (*Escherichia coli*). Antifungal assays were evaluated using *Candida albicans*, *Penicillium chrysogenum*, *Aspergillus fumigatus*, and *Aspergillus niger*. These microbes were chosen primarily on the basis of their importance as pathogens of humans. Pure cultures of all experimental bacteria and fungi were American Type Culture Collection (ATCC), obtained from Total Laboratory, South Africa.

Nutrient agar and Sabouraud dextrose agar (SDA) media were prepared and poured into sterilised disposable petri dishes under aseptic conditions according to the recommendations of the manufacturer. The plates were labeled and inoculated with 100 µL of 0.5 Mcfarland solutions of the respective organisms, and loaded with extract of *E. golondrina* (50 mg/mL) into 6 mm wells. Amoxicillin and Amphotericin B (25–50 μg/well) were used as positive controls for bacteria and fungi respectively. Each test was replicated three times. After proper incubation period at 37 °C, zones of inhibition were recorded in millimeters. The microdilution method was employed to determine the minimum inhibitory concentration (MIC) of the plant extracts using 96 well microtitre plates as previously described (Otang et al. 2012). The smallest concentration of the plant extract that was able to kill the microorganisms was considered as the minimum inhibitory concentration (MIC).

### Antioxidant assays

#### Assay of DPPH scavenging activity

The DPPH (1,1-diphenyl-2-picrylhydrazyl) radical-scavenging activity of the test extracts was examined as previously described (Ebrahimzadeh et al. 2010). Different concentrations (0.025–0.5 μg/mL) of each extract were added, at an equal volume, to methanolic solution of DPPH (100 μM). The mixture was kept in the dark for 30 min. Vitamin C and Rutin were used as standard controls and three replications were made. After 30 min, the absorbance (A) was measured at 518 nm and converted into the percentage antioxidant activity using the following equation: % Scavenged [DPPH] = [(Ao − A1)/Ao] × 100, where Ao was the absorbance of the control and A1 was the absorbance of extract and standard.

#### Assay of nitric oxide scavenging activity

This assay was done according to the procedure of Ebrahimzadeh et al. (2010). Two mL of 10 mM sodium nitroprusside in 0.5 mM phosphate-buffered saline (pH 7.4) was mixed with different concentrations of the acetone extract of *E.golondrina* dissolved in water and incubated at 25 °C for 2.5 h. After the incubation period, 0.5 mL of Griess reagent was added and the absorbance was read at 540 nm. The Inhibition of nitric oxide radical generation was measured by comparing the absorbance value of the controls (Vitamin C and Rutin) with that of the test solution.

#### Reducing power assay

Different concentrations (0.025–0.05 μg/mL) of the acetone extract of the plant in distilled water were mixed with 2.5 mL of 0.2 M phosphate buffer (pH 6.6) and 2.5 mL potassium ferricyanide (1 % w/v). The mixture was then incubated at 50 °C for 20 min, and 2.5 mL of trichloroacetic acid (10 % w/v) was added. The mixture was centrifuged at 3000 rpm for 10 min. 2.5 mL of the supernatant was mixed with an equal volume of distilled water and 0.5 mL of FeCl_3_ (0.1 % w/v) and the absorbance was measured at 700 nm. Vitamin C and Rutin were used as positive controls.

### ABTS cation free radical-scavenging activity

For ABTS (2,2′-azino-bis 3-ethylbenzothiazoline-6-sulphonic acid) assay, the procedure followed was the method of Zheleva-Dimitrova et al. (2010) and Roberta et al. (1999) with some modifications. ABTS was dissolved in water to make a concentration of 7 mmol/L. ABTS^+^ was produced by reacting the ABTS stock solution with 2.45 mmol/L potassium persulfate (final concentration) and the mixture was left in the dark at room temperature for 12–16 h before use. The ABTS^+^ stock solution was diluted with 80 % methanol to an absorbance of 0.70 ± 0.02 at 734 nm. 4.85 mL of diluted ABTS ^+^ was added to 0.15 mL of tenfold diluted samples (final concentrations 0.025–0.5 μg/mL of each extract were added, at an equal volume of dry material). The absorbance reading was taken at 6 min after the initial mixing. Gallic acid and BHT were used as positive controls. The activities of the samples were evaluated by comparison with a control (containing 4.85 mL of ABTS solution and 0.15 mL of 45 % methanol). ABTS ^+^ scavenging activity was calculated by the following formula:

$${\text{ABTS}}^{ + } \,{\text{scavenging}}\,{\text{activity}}\,(\% ) = [(Ac - As)/Ac] \times 100$$

where *A*_C_ is the absorbance value of the control and *A*_S_ is the absorbance value of the added samples test solution.

### GC–MS analysis

Five hundred grams (500 g) of the plant sample was distilled using distilled water in an all-glass Clevenger apparatus in accordance with British Pharmacopoeia (1980). Heat was supplied to the heating mantle (50 °C) and the essential oils were extracted with 3 L of distilled water for 3 h. The oil collected was analysed using gas chromatograph-mass spectrometry (GC–MS). The GC–MS was carried out using Agilent 7890B GC system coupled to an Agilent 5977A MSD with a Zebron-5MS column (ZB-5MS 30 m × 0.25 mm × 0.25 µm) (5 %-phenylmethylpolysiloxane). GC grade helium was used as a carrier gas at a flow rate of 2 mL/min and splitless 1 µL auto-injections were used. The injection temperature was 250 °C and source temperature was 250 °C. Oven temperature was 70 °C, ramp 15 °C/min to 120 °C, ramp at 10 °C/min to 180 °C then ramp at 20 °C/min to 270 °C and hold for 3 min. Data was gathered with Chem station. The oil components were identified by matching their mass spectra and retention indices with those of the Wiley 275 library (Wiley, New York) in the computer library and literature (Siegler 1998). The yield of the oil was calculated per gram of the plant material, while the percentage composition was calculated from summation of the peak areas of the total oil composition.

### Statistical analysis

The zones of inhibitions induced by the plant extract against the tested microbes were given as mean ± standard deviation of 3 replicates. Experimental results were analyzed by SPSS version 16.0 (SPSS Inc. Chicago, IL). Differences between means were determined using one-way ANOVA and least significant difference test. The level of statistical significance was set at *P* ≤ 0.05.

## Results

### Antimicrobial activity

**Fig. 1:**
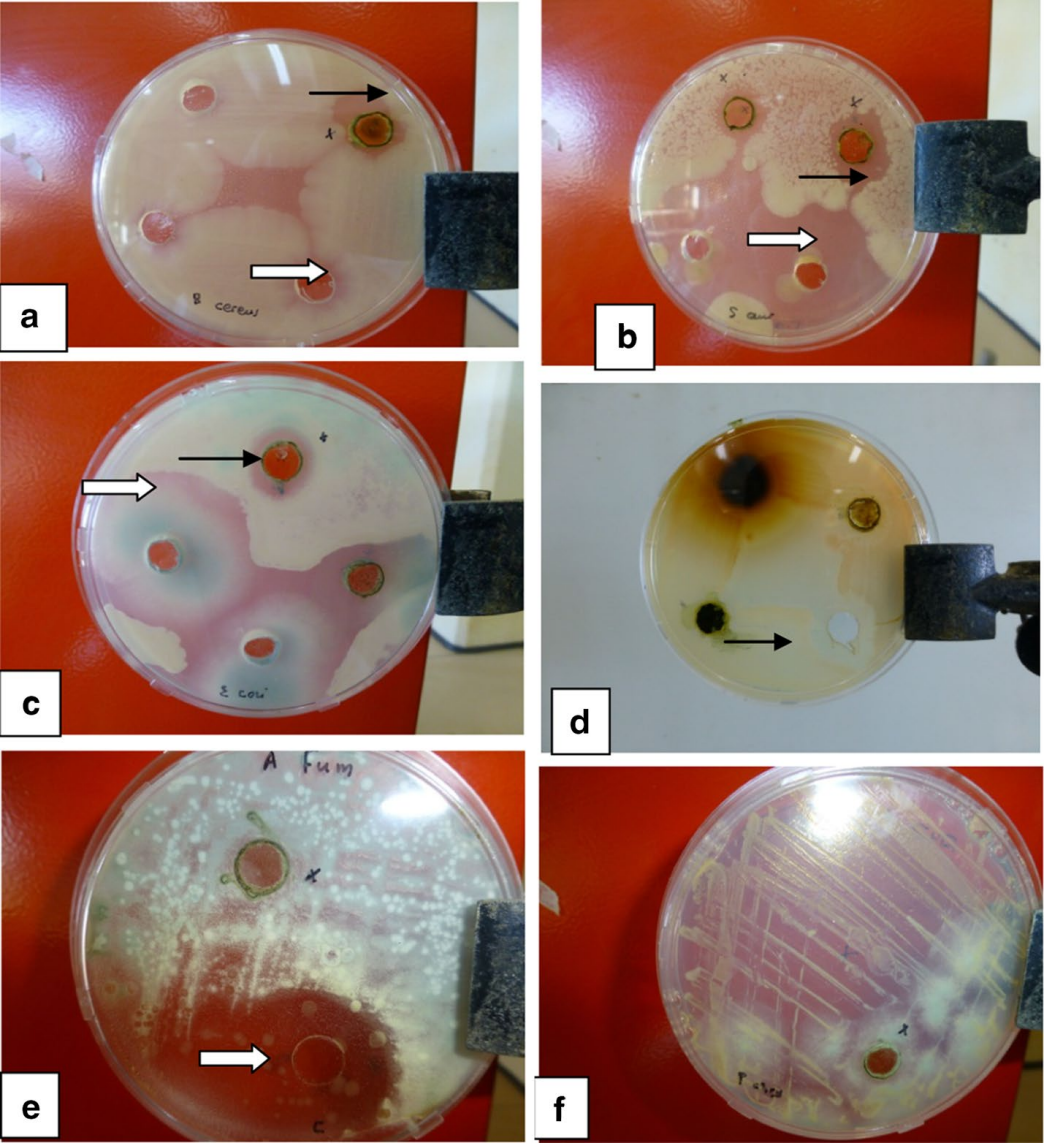
Susceptibility/tolerance of selected bacteria (**a** *B. cereus*; **b** *S. aureus*; **c** *E. coli*) and fungi (**d** *C. albicans*; **e** *Aspergillus fumigates*; **f** *P. chrysogenum*) to the acetone extract of *Euphorbia golondrina* L.C. Wheeler. *Black arrow* indicates zone of inhibition by extract, *white arrow* indicates zone of inhibition by positive control

The extraction yield was 9.3 %. The results of the agar well diffusion assay and the MICs of the acetone extract of *E. golondrina* are summarised in the Table 1 and Fig. 1 below. The highest antibacterial activity with inhibition zone diameter of 20 ± 1.1 mm, MIC value of 0.01 mg/mL was observed against *B. cereus*, followed by *S. aureus* with inhibition zone diameter of 17 ± 1.6 mm, MIC value of 2.5 mg/mL, which was significantly lower (P < 0.05) than the positive control. None of the fungi was inhibited by the acetone extract of *E. golondrina* except *C. albicans* wherein the zone of inhibition (21 ± 2.1 mm) was not significantly different from that of the positive control.

**Table 1:** Inhibition of microbial growth by acetone extract of *Euphorbia golondrina* L.C. Wheeler

Microorganism	Zone of inhibition (mm)		Minimum inhibition concentration (mg/mL)	
	Extract	Positive control	Extract	Positive control
Bacteria				
*Escherichia coli*	15 ± 1.1	30 ± 0.1	2.5	<0.01
*Enterococcus faecalis*	15 ± 1.6	31 ± 1.1	2.5	<0.01
*Staphylococcus aureus*	17 ± 1.6	32 ± 3.1	2.5	<0.01
*Bacillus cereus*	20 ± 1.1	35 ± 0.1	0.01	<0.01
Fungi				
*Penicillium chrysogenum*	Na	29 ± 4.1	Na	<0.01
*Aspergillus fumigatus*	Na	28 ± 2.1	Na	<0.01
*Aspergillus niger*	Na	26 ± 0.8		<0.01
*Candida albicans*	21 ± 2.1*	22 ± 0.8	0.01	<0.01

Values are mean ± SD of triplicates experiments, *Na* not active

* Not significantly different from positive control (P < 0.05), positive control: Amphotericin B for fungi and amoxicillin for bacteria

### Results of antioxidant assays

#### DPPH radical-scavenging activity

**Fig. 2:**
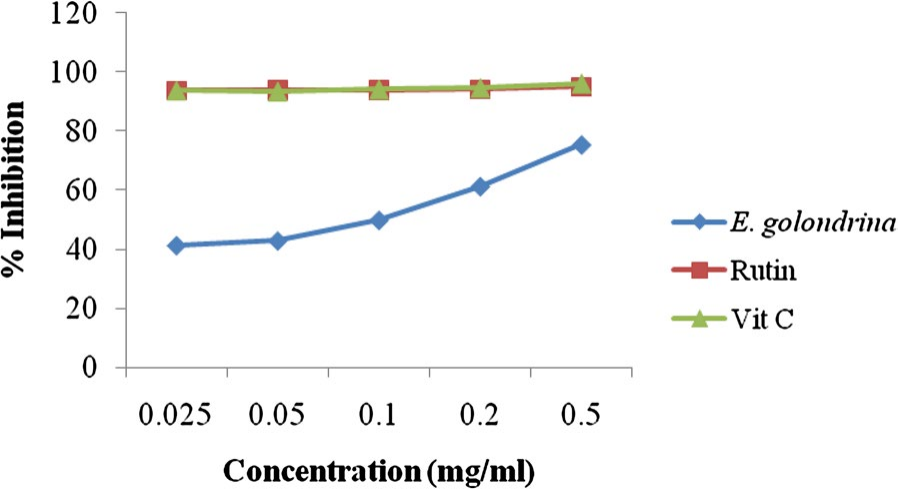
DPPH scavenging activity of the acetone extract of *Euphorbia golondrina* L.C. Wheeler

The results of the DPPH assay showed that the scavenging activity of Vitamin C and Rutin were higher than *E. golondrina* at all concentrations (Fig. 2). However, the DPPH scavenging activity of *E. golondrina* increased with increasing concentration.

#### Nitric oxide scavenging activity

**Fig. 3:**
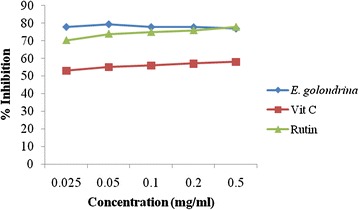
Nitric oxide scavenging activity of the acetone extract of *Euphorbia golondrina* L.C. Wheeler

The nitric oxide (NO) scavenging activity of *E. golondrina* was not dose-dependent, but higher than those of both Rutin and Vitamin C (Fig. 3).

#### Reducing power assay

**Fig. 4:**
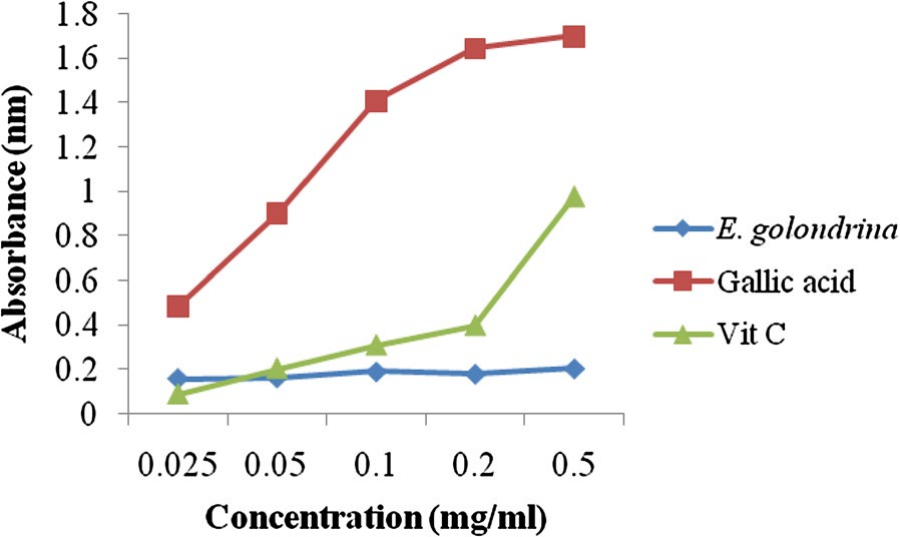
Reducing power of the acetone extract of *Euphorbia golondrina* L.C. Wheeler

The dose–response curve for the reducing powers of the acetone extract of *E. golondrina* (as indicated by the absorbance at 700 nm) is shown in Fig. 4. Increased absorbance indicates increased reducing power. The reducing power of the acetone extract of *E. golondrina* was lower than those of both controls.

#### ABTS scavenging activity

**Fig. 5:**
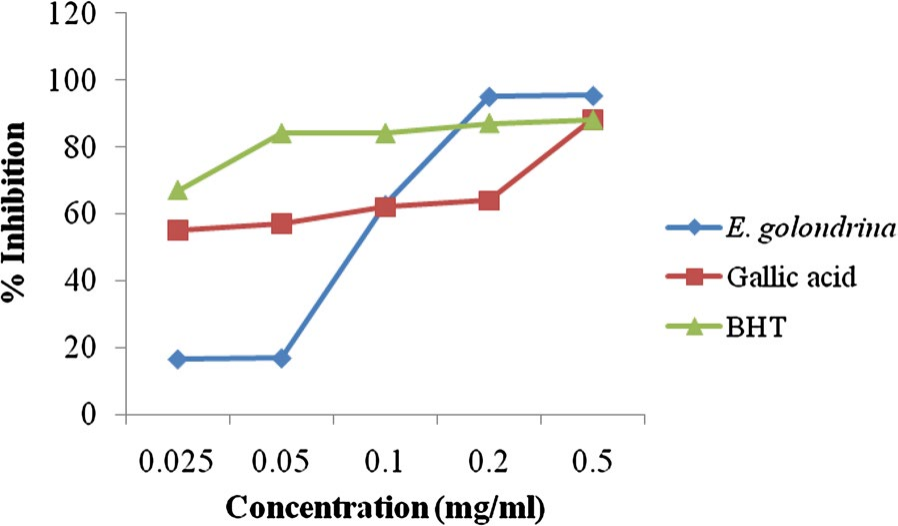
ABTS scavenging activity of the acetone extract of *Euphorbia golondrina* L.C. Wheeler

Scavenging activity was expressed as percentage of inhibition of ABTS^+^ free radical (Fig. 5). The ABTS scavenging activity of *E. golondrina* was higher than that of gallic acid and BHT at concentrations greater than 0.1 and 0.2 mg/mL respectively.

### Result of the GC–MS analysis of *E. golondrina*

The characteristics of the phytochemicals identified, including the retention time (RT), relative peak area percentage (peak area relative to the total peak area) and chemical structures of phytoconstituents of the *E. golondrina* extract are summarized in Table 2. The various classes of phytochemicals in the *E. golondrina* plant provided the antioxidant and antimicrobial potency of the plant. Thirty compounds were identified from the steam distilled volatile of *E. golondrina* with caryophyllene oxide (14.16 %), camphor (9.41 %) and phytol (5.75 %) being the most abundant. These were followed by 2,6-diisopropylnaphthalene (4.77 %), octasiloxane (4.38 %), furan (3.04 %), nanonal (3.32 %), eucalyptol (2.92 %) and 3,4-dimethylanisole (2.30 %).

**Table 2:**
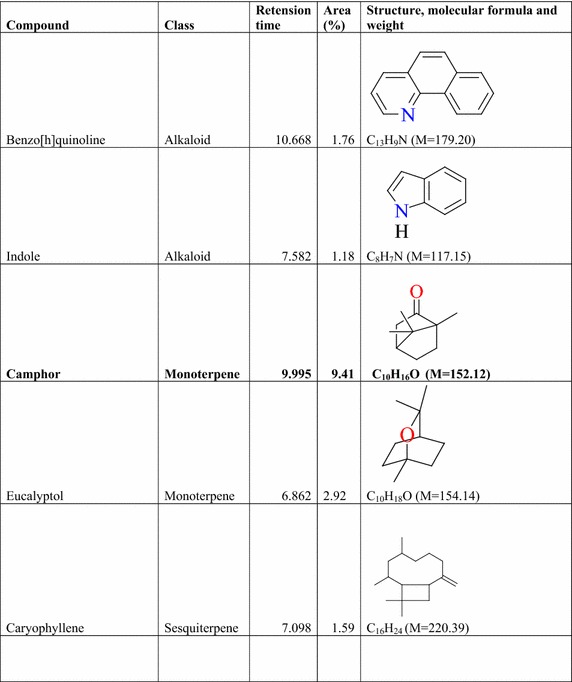

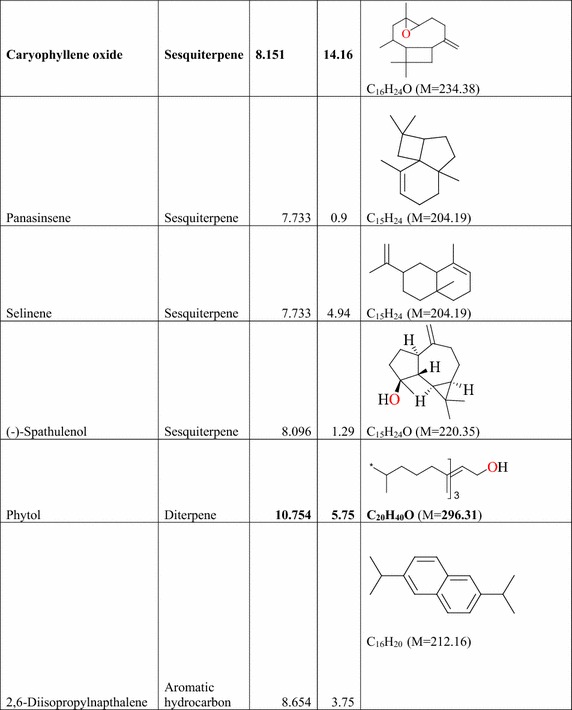

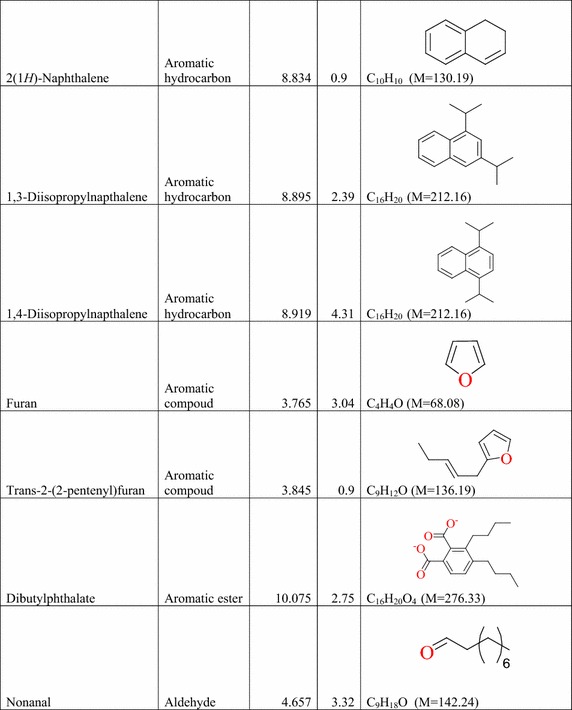

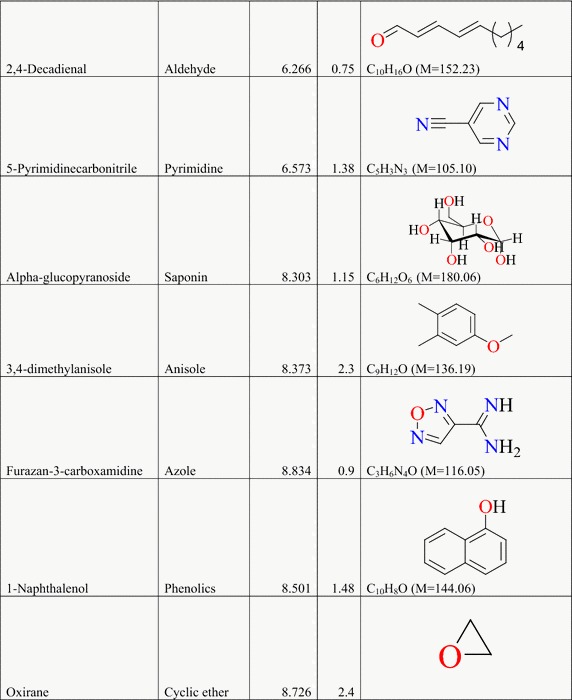

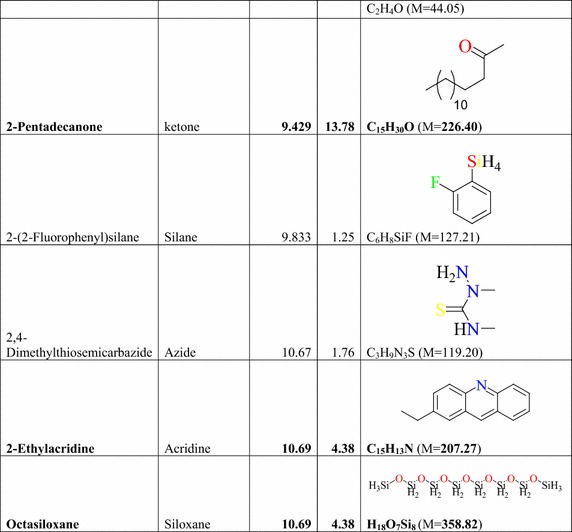
Phytochemicals in *Euphorbia golondrina* L.C. Wheeler identified by GC–MS

## Discussion

*Euphorbia golondrina* is used to treat enterocolitis, vaginitis and conjunctivitis by the Mexicans and the Mundani people of the mount Bambouto Caldera, SouthWestern Cameroon (Ndam et al. 2015a, b; Rodriguez 2013; Aleksandroff 2011). Vaginitis is responsible for an estimated 10 million physician visits by women annually and invasion of the epithelial cells of female genitals by *C. albicans* causes itching and inflammation (Gibbs et al. 2009). Treatment by azoles such as fluconazole has not been effective in eradicating these infections. Hence, many women use complementary treatments (Imhof et al. 2005) with alternative medicine partly because of their fewer side effects and lower cost. The fact that the acetone extract of *E. golondrina* was fungicidal against *C. albicans*, is a partial justification of the ethnopharmacological use of the plant against vaginitis and also highlights the possibility of discovering new antifungal compounds with novel mechanisms of action from the plant. However, the inability of the acetone extract of *E. golondrina* to inhibit the growth of *P. chrysogenum*, *A. fumigates*, and *A. niger* despite its extensive use in traditional medicine is worth investigating. We hypothesise that acetone may not have extracted some of the antifungal compounds present in the plant or the medicinal compounds in the plant may have alternative modes of therapeutic action that may boost the immune system of the body.

Enterocolitis is an inflammation of the digestive tract that results in enteritis of the small intestine and colitis of the colon. Many bacteria as well as fungi and viruses are responsible for the development of enterocolitis in humans but the most common etiologic agents of the disease are: *Salmonella*, *Shigella*, *E. coli*, and *S. aureus*. Although most *E. coli* are commensals found in the gut of humans, some pathogenic strains are distinguished from normal flora by the possession of virulence factors such as exotoxins. The most common symptoms of enterocolitis are frequent diarrhoea, fever, vomiting and abdominal pain. *S. aureus* is the causative agent of acute bacterial conjunctivitis in humans, where as *Streptococcus pneumoniae* and *Haemophilus influenzae* are causative agents of conjunctivitis in children (Buznach et al. 2005). In the current study, the acetone extract of *E. golondrina* displayed a moderate antibacterial activity especially against *E. coli*, strong antibacterial activity against *S. aureus* and *B. cereus*, indicating that the plant could be a good source of antibacterial and confirming its ethnomedicinal usage in the study area against enterocolitis and conjunctivitis. Although there have been no previous reports on the antibacterial activity of *E. golondrina* in scientific literature, other *Euphorbia* species have been noted for their antibacterial activity. The ethyl acetate fraction of the methanolic extract of *Euphorbia pulcherrima* was reported to contain phytochemicals which showed remarkable activities against *E. coli*, *S. aureus*, *Salmonella typhi*, and *Pseudomonas aeruginosa* (Sharif et al. 2011). Methanolic extracts and latex of some species of *Euphorbia* are known to inhibit the growth of *S. aureus*, *Bacillus megaterium*, *Proteus vulgaris*, *Klebsiella pneumonia*, *E. coli*, *P. aeruginosa* and *C. albicans* (Kirbag et al. 2013).

In the Mexican ethnopharmacopoeia, *E. golondrina* is used to treat rheumatism while it is applied to relieve painful swellings by the Mundani people of the mount Bambouto Caldera, SouthWestern Cameroon (Ndam et al. 2015a, b; Rodriguez 2013; Aleksandroff 2011). Rheumatism is an inflammatory autoimmune disease marked by polyarthritis that is erosive, progressive and chronic. The use of *E. golondrina* for the traditional management of rheumatism in Cameroon suggests that antioxidants in the plant may have an important role to play as they lower the oxidative stress and the resultant inflammatory damage. In the present study, antioxidant evaluation of the acetone extract of *E. golondrina* was conducted using DPPH, reducing power, nitric oxide and ABTS radical scavenging assays. Potent antioxidant activity was observed during the nitric oxide and ABTS scavenging assays. The ABTS radical (ABTS^·+^) scavenging abilities of the acetone extract of *E. golondrina* were more effective than the standards; gallic acid and BHT at higher concentrations of 0.1 and 0.2 mg/mL, respectively. It was assumed that antioxidants in *E. golondrina* simply reduce the radical back to the parent substrate, ABTS. The radical scavenging activity of the crude extract of *Euphorbia cortinifolia* against ABTS showed IC_50_ of 95.61 ± 1.5 µg/mL, compared to the standard, BHT with IC_50_ of 90.91 ± 1.4 µg/mL (Hosain et al. 2014). Whilst there exist a dearth of scientific literature on the ABTS scavenging activity of *Euphorbia* species, this study present the first report on the ABTS scavenging activity *of E. golondrina*.

In the present study, the acetone extract of *E. golondrina* at different concentrations was assessed for nitrite free radical scavenging activity in an in vitro model. The nitric oxide scavenging activity of the acetone extract of *E. golondrina* was higher than that of both vitamin C and Rutin. The activity of the acetone extract could be attributed to phyto-components such as phenolic compounds. Polyphenols are major antioxidant components present in most plant extracts (Ebrahimzadeh et al. 2010). The acetone extract is thought to contain polyphenols since acetone effectively penetrates cellular membranes of plants resulting in the extraction of polyphenols (Sumazian et al. 2010). Other studies have shown that the genus *Euphorbia* is extremely rich in polyphenolics; 9 antioxidant polyphenols: scopoletin, scoparone, isoscopoletin, quercetin, isorhamnetin, pinocembrin, kaempferol, luteolin, and gallic acid were preliminary identified from *Euphorbia hirta* (Wu et al. 2012). Ferulic acid is a phenolic compound that was identified from *Euphorbia tirucalli* (De Araújo 2014). Boudiar et al. (2010) identified six known flavonoids namely, kaempferol, kaempferol 3-*O*-glucoside, kaempferol 3-rutinoside, quercetin, quercetin 3-*O*-glucoside, and rutin from the aerial parts of *Euphorbia guyoniana*. This suggests that *E. golondrina* might contain compounds that scavenge and this may account for the regulation of pathological conditions induced by nitric oxide and its oxidation product, peroxynitrite. Nitric oxide has been reported to be scavenged by flavonoids and saponins (Patel et al. 2010). Hence, the presence of phenolics and saponins compounds such as 1-naphthalenol and alpha-glucopyranoside could partially explain why *E. golondrina* is more potent at quenching the nitric oxide radical. Since chronic exposure to nitric oxide radical is associated with inflammatory diseases such as arthritis, the scavenging of *E. golondrina* acetone extract could partially justify the folkloric use of the plant in the treatment of rheumatism.

The GC–MS analysis of *E. golondrina* reveals an extremely impressive array of compounds such as caryophyllene oxide, eucalyptol, dibutylthalate, acetanilide, phenylacetamide, phytol, camphor, etc. Caryophyllene, selinene, paninsene and spathulenol are the sesquiterpenes identified in *E. golondrina*. Terpenoids are active against bacteria and fungi (Salari et al. 2006; Cowan 1999). Eucalyptol showed antibacterial activity against some pathogenic bacteria in the respiratory tract (Salari et al. 2006) and because of its antimicrobial properties, it is also used in dental care and soaps. Dibutylphthalate was the only phthalate that was identified from *E. golondrina.* Although few reports are available for the antibacterial potential of phthalate derivatives from plants, bis (2-ethylhexyl) phthalate extracted from *Streptomyces bangladheshiensis* has been reported to show antibacterial activity against Gram-positive bacteria while the anti-inflammatory activity of di (2-ethylhexyl) phthalate from *Alchornea* sp. was reported (Camila et al. 2013). Phenylacetamide (acetanilide), although no longer used as a drug, it was the first analgesic and antipyretic aniline and the success of its metabolite is well documented (Bertolini et al. 2006). The in vitro antioxidant effect of phytol including its capacity to remove hydroxyl radicals and nitric oxide has also been reported (Camila et al. 2013). Camphor is commonly used topically to relieve muscular pains especially in osteoarthritis and also for the treatment of fungal infections of the toe nail (Farhat et al. 2001). In the current study, structural characterisation of the purported compounds was limited to comparative MS spectral analysis of the steam distilled volatiles of *E. golondrina.* Further, structural characterisation based on ^1^H and ^13^C NMR is required to demonstrate structural integrity including correct stereochemistry which may be present in terpene compounds.

## Conclusion

This study has demonstrated the activity of the acetone extract of *E. golondrina* against microorganisms of human pathogenic interest which confirms the ethnopharmacological uses of the plant. However, it was not active against *Penicilium* and *Aspergilus* which are important human pathogens and storage pathogens of plant and plant products. The extract also showed significant antioxidant properties, indicative of its potential as a source. The plethora of compounds elucidated by the GC–MS needs further investigation for possible exploitation for pharmaceutical uses.
